# Methionine aminopeptidase 2 is a key regulator of apoptotic like cell death in *Leishmania donovani*

**DOI:** 10.1038/s41598-017-00186-9

**Published:** 2017-03-07

**Authors:** Ritesh Kumar, Kartikeya Tiwari, Vikash Kumar Dubey

**Affiliations:** Department of Biosciences and Bioengineering, Indian Institute of Technology Guwahati, Assam, 781039 India

## Abstract

We investigate the role of methionine aminopeptidase 2 (MAP2) in miltefosine induced programmed cell death (PCD) in promastigote form of *L. donovani*. We report that TNP-470, an inhibitor of MAP2, inhibits programmed cell death in miltefosine treated promastigotes. It inhibits the biochemical features of metazoan apoptosis, including caspase3/7 protease like activity, oligonucleosomal DNA fragmentation, collapse of mitochondrial transmembrane potential, and increase in cytosolic pool of calcium ions but did not prevent the cell death and phosphatidyl serine externalization. The data suggests that the MAP2 is involved in the regulation of PCD in parasite. Moreover, TNP-470 shows the leishmanicidal activity (IC_50_ = 15 µM) and *in vitro* inhibition of *Ld*MAP2 activity (*K*_*i*_ = 13.5 nM). Further studies on MAP2 and identification of death signaling pathways provide valuable information that could be exploited to understand the role of non caspase proteases in PCD of *L. donovani.*

## Introduction

Apoptotic like cell death in *Leishmania* and other unicellular organisms after drug treatment or under stress conditions is well characterized^1–5^. The major biochemical features of apoptosis include the proteolytic activation of caspase3/7 like proteases^6, 7^, permeabilization of mitochondrial membrane resulting in changes in transmembrane potential, release of cytochrome C from mitochondria^8^, oligonucleosomal DNA fragmentation^9^ and increase in Annexin-V positive cells^10^. *Leishmania* parasite shows all these characteristics under apoptotic conditions^3–5^ *.* Caspase3/7 protease like activities associated with apoptotic cell death in *Leishmania* are also very well documented^2–5^. However, no caspase gene or caspase homologue has been identified in *Leishmania* genome^11^. The genes encoding metacaspases which belongs to an ancestral matacaspase, paracaspase and caspase superfamily have been identified in several lower eukaryotes including *Leishmania* ^11–13^. Very few reports have been published on the role of metacaspases in programmed cell death (PCD) in *Leishmania*. There are certain reports that point towards the role of metacaspases in the regulation of apoptotic like cell death in *L. major* ^14–16^ *.* A detailed biochemical characterization of metacapases in *Trypanosoma* and *Leishmania* signifies that it prefers the substrate specificity with an Arginine and Lysine residue at P1 position and are unable to cleave caspase specific substrates as well as insensitive to caspase specific inhibitors^17, 18^. Thus, the source of caspase like activity in apoptotic stage *L. donovani* promastigotes cell lysate remains elusive. Further, role of other proteases in apoptotic like cell death in *Leishmania* is also not yet extensively studied. Miltefosine, the only available oral drug against the parasite, is known to induce apoptosis in *L. donovani* promastigotes^19^. The treatment of *L. donovani* promastigote cells with miltefosine induced the over-expression of methionine aminopeptidase 2 (*MAP2*) by 3.5 times compared to control cells^20^. This finding suggests the involvement of MAP2 in apoptosis like cell death of the parasite and prompted us to study the role of MAP2 in the apoptotic processes of *Leishmania* parasite.

Methionine aminopeptidase (MAP) catalyzes the removal of N-terminal methionine residue during translation of protein^21^. Removal of methionine residue from newly synthesized protein is important for proper translocation of protein. Two types of methionine aminopeptidases are reported in eukaryotes, methionine aminopeptidase 1 (MAP1) and methionine aminopeptidase 2 (MAP2). MAP2 is also involved in the protection of eukaryotic initiation factor 2 alpha (eIF2-α) from inhibitory phosphorylation^22–25^. Several publications have suggested the role of MAP2 in the angiogenesis *i.e.* the formation of new blood vessels in higher eukaryotes which is necessary for tumor growth and metastasis^26, 27^. Increased expression of MAP2 is reported in mesothelioma cells and several other cancer cells^28^. Compounds belonging to fumagilin family are potent inhibitors of angiogenesis, and are reported to bind MAP2 and inhibit its activity^29^. An analog of fumagilin, TNP-470, is reported to inhibit MAP2 selectively without inhibiting closely related isoenzyme MAP1^30^. However, the functional role(s) of MAP2 in protozoan parasite *Leishmania* is not very well explored.

In the present study, we cloned, expressed, purified and characterized MAP2. Further, we confirmed inhibition of MAP2 from *L. donovani* (*Ld*MAP2) by TNP-470 and derived biochemical parameters of inhibition. We have successfully demonstrated that a specific MAP2 inhibitor prevents miltefosine induced apoptotic features like increased caspase3/7 protease like activity, DNA degradation, disruption in transmembrane potential of mitochondria, and increase in cytosolic calcium.

## Results

### Cloning, Expression, Purification and Biochemical characterization of *Ld*MAP2

Various steps of cloning, expression and purification of *Ld*MAP2 are shown in (Fig. S1). The genomic DNA of *L. donovani* was amplified using gene specific primers of *Ld*MAP2. The amplified band of 1.39 kb was cloned in pET-28a(+) vector as described in methods section. The pET-28a(+)-*Ld*MAP2 construct was confirmed by PCR and restriction digestion. The PCR amplification of 1.39 kb band and release of 1.39 kb band by double digestion with *Eco*RI and *Xho*I, confirmed the insertion of *Ld*MAP2 in pET-28a(+) vector (Fig. S1). The clone was further confirmed by sequencing using primers for T7 promoter and T7 terminator.

The pET-28a(+)-*Ld*MAP2 construct was transformed into BL21 (DE3) expression strain of *E. coli*, induced by 250 μM final concentration of IPTG (25 °C, 8 hrs) for the overexpression of protein and produced an insoluble and enzymatically inactive protein in inclusion bodies. The recombinant protein from inclusion bodies was solubilized in 8 M urea and purified by Ni-NTA affinity chromatography. The purified protein was dialyzed, refolded using the refolding buffer system as described in the methods section, and run on SDS-PAGE gel. Migration of *Ld*MAP2 on SDS-PAGE showed an apparent molecular mass of 55.1 kDa (Fig. S1). Western blot analysis using mouse anti-His antibodies recognized the recombinant *Ld*MAP2 containing His-tag at its N-terminal (Fig. S1). The approximate yield of recombinant *Ld*MAP2 protein was 5 to 7 mg/L.

**Figure 1:**
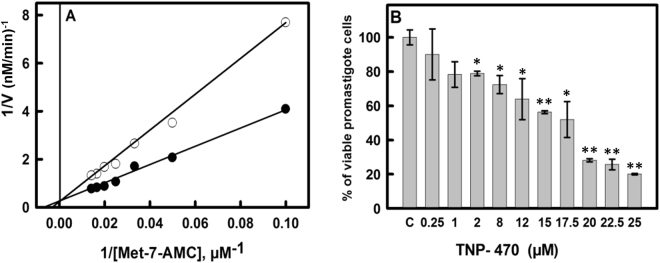
Lineweaver-Burk plot and Cell proliferation assay in presence of MAP2 inhibitor, TNP-470. (**A**) Inhibition studies for TNP-470 (100 µM), Competitive inhibition with respect to Met-AMC as a substrate. *Ki* value was found to be 13.5 nM. (**B**) MTT Assay; Effect of TNP-470 on *L. donovani* promastigotes, IC_50_ value against *L. donovani* promastigotes were found to be 15.01 ± 0.73 µM. Data represents the mean ± SD of three independent experiments**.** Statistical analysis was done using Student’s unpaired t-test in SigmaPlot software (*denotes p value ≤ 0.05 and **denotes p value < 0.01).

The *Ld*MAP2 enzymatic assay was carried out by measuring the release of 7-AMC by fluorescence at an excitation and emission wavelength of 360 nm and 440 nm, respectively. A pH range (pH 4.0 to 10.0) in mixed buffer system as mentioned in the methods section was used to determine the optimum pH for the enzyme activity against the fluorogenic substrate Methionine-7-amido-4-methyl coumarin (Met-AMC). The temperature range (20 °C to 60 °C) was used to check the optimum temperature. The pH and temperature optima studies showed an optimum pH of 7.5 and temperature optima at 37 °C for the substrate Met-AMC (Fig. S2). The activity of purified *Ld*MAP2 was reduced to ~5% after incubating with 5 mM EDTA, signifying that metal ions are required for enzyme activity. The activity was found to be maximum in the presence of Ni(II), followed by Cu(II), Ca(II), Mg(II), Zn(II) and Mn(II). Furthermore, no *Ld*MAP2 activity was observed in the absence of divalent metal ions (Fig. S2). The enzyme kinetic studies showed the *Km* and *Vmax* values of 0.2 mM and 5.71 nM/min, respectively (Fig. 1A). The *Km* and *Vmax* values were calculated by varying the substrate concentration of Met-AMC (10 µM to 100 µM).

### Inhibition studies of functionally active *Ld*MAP2 by TNP-470 and leishmanicidal activity of the inhibitor on *L. donovani* promastigotes

The inhibitor TNP-470 was found to inhibit *Ld*MAP2 activity, which was assessed by Lineweaver-Burk plots (Fig. 1A). TNP-470 showed a competitive mode of inhibition at a final concentration of 100 µM with respect to varied concentration of Met-AMC as *Vmax* did not change but there was an increase in *Km* value in the presence of inhibitor. The inhibitory constant (*Ki*) value was found to be 13.5 nM as the substrate concentration was varied.

*L. donovani* promastigote cells (2.5 × 10^6^ cells/mL) were treated with varying concentrations (0.25 µM to 50 µM) of TNP-470 for 24 h to check the antileishmanial activity. TNP-470 showed significant leishmanicidal activity with IC_50_ values of 15.01 ± 0.73 µM (Fig. 1B). Promastigote cells treated with 0.2% DMSO were used as negative control whereas cells treated with 25 µM of miltefosine served as positive control.

### TNP-470 causes inhibition of Caspase3/7 protease like activity and oligonucleosomal-DNA fragmentation in *L. donovani*

**Figure 2:**
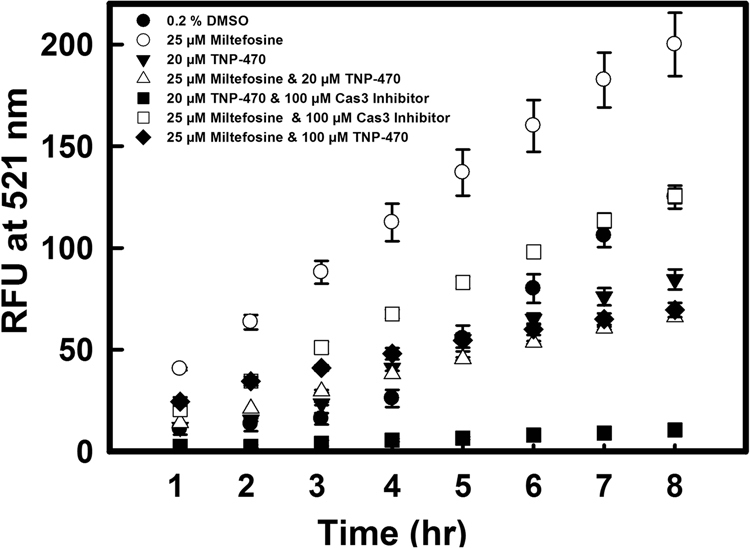
Activation of Caspase-3/7 like proteases inside *L*. *donovani* promastigote cells. Control cells (0.2% DMSO), promastigotes induced with 25 µM of miltefosine, and by 20 µM TNP-470, alone or both. TNP-470 induced and miltefosine induced cell lysates were incubated with 100 µM of Cas3 inhibitor for 30 min, Miltefosine treated cell lysates were also incubated with 100 µM of MAP2 inhibitor. Data represents the mean ± SD of three independent experiments.

Treatment of *L. donovani* promastigotes with miltefosine strongly revealed the apoptosis like mode of cell death with activation of Cas3/7 protease like activity which is well documented by various groups^31, 32^. In control studies, promastigote cells treated with miltefosine (25 µM for 18 h incubation at 25 °C) also showed an increased Cas3/7 protease like activity whereas the promastigote cells treated with TNP-470 (20 µM for 18 h incubation at 25 °C) did not show activation of Cas3/7 protease like activity. Unlike miltefosine treated cells, no significant increase in activity of Cas3/7 like protease was observed in case of promastigotes treated with both miltefosine (25 µM, 18 h) and TNP-470 (20 µM, 18 h). Cas3/7 protease like activity in cell lysates of miltefosine (25 µM) treated cells in presence of 100 µM of caspase-3 inhibitor (N-Acetyl-Asp-Glu-Val-Asp-al) or in presence of TNP-470 (20 µM) was even lesser than control cells (Fig. 2). Control cells treated with 0.2% DMSO did not show significant increase in Cas3/7 protease like activity.

**Figure 3:**
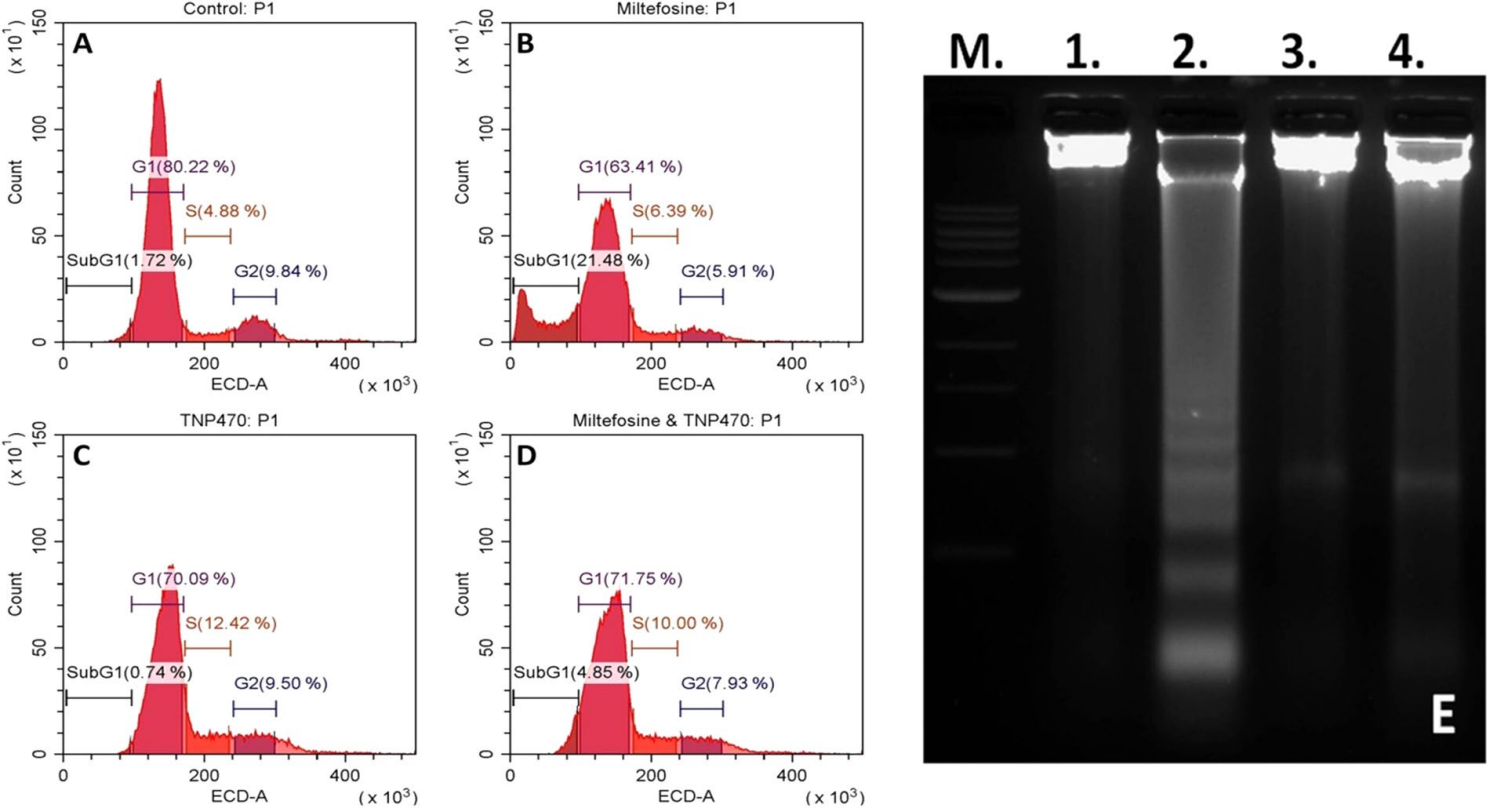
Cell cycle and DNA fragmentation analysis of *L. donovani* promastigotes. (**A**) Control promastigotes treated with 0.2% DMSO. (**B**) Promastigotes induced with 25 µM of miltefosine. (**C**) Cells treated with 20 µM of TNP-470. (**D**) Miltefosine induced promastigotes treated by TNP-470. (**E**) *L. donovani* genomic DNA fragmentation assay. Lane M. 1 kb ladder, Lane 1. Control cells (0.2% DMSO), Lane 2. Miltefosine induced promastigotes showing fragmentation of genomic DNA, Lane 3. TNP-470 treated, showing no fragmentation of genomic DNA, Lane 4. Both miltefosine and TNP-470 treated cells (showing inhibition of genomic DNA fragmentation).

Flow cytometric analysis of promastigotes population was used to calculate the percentage of sub-G_**1**_cells (hypo-diploid cells) after cell permeabilization and staining with propidium–iodide (PI). The fluorescence intensity of bound PI correlates the amount of DNA in which the percentage of sub-G_1_ peak shows the amount of DNA degradation in apoptotic like cells. Only 1.72% of the control cells (promastigote cells treated with 0.2% DMSO) were found in the sub-G_1_ region (Fig. 3A) whereas leishmanial cells treated with 25 µM of miltefosine for 12 h resulted in 21.48% cells in apoptotic region (sub-G_1_ peak region) (Fig. 3B). In contrast, no sub-G_1_ peak region was found in TNP-470 (20 µM for 12 h) treated promastigote cells (Fig. 3C). Promastigote cells treated with both miltefosine and TNP-470 for 12 h resulted in only 4.85% cells in sub-G_1_ peak region (Fig. 3D), thus suggesting that MAP2 inhibitors prevents the induction of nuclear apoptosis like features in *L. donovani.*

Fragmentation of genomic DNA into nucleosomal units is considered as the characteristic feature of apoptotic cell death^33^. DNA fragmentation analysis of promastigotes treated with miltefosine (25 µM), showed fragmentation of genomic DNA on agarose gel electrophoresis as reported earlier^31, 32^. No significant fragmentation of genomic DNA on agarose gel electrophoresis was observed in promastigote cells treated with TNP-470 (20 µM) and in control cells treated with 0.2% DMSO. Interestingly, the promastigote cells treated with both miltefosine (25 µM) and TNP-470 (20 µM) for 18 h did not show fragmentation of genomic DNA into oligonucleosomal fragments, suggesting the inhibition of DNA fragmentation in miltefosine mediated PCD (Fig. 3E).

### TNP-470 treatment of *L. donovani* promastigotes does not inhibit the phosphatidyl serine externalization

**Figure 4:**
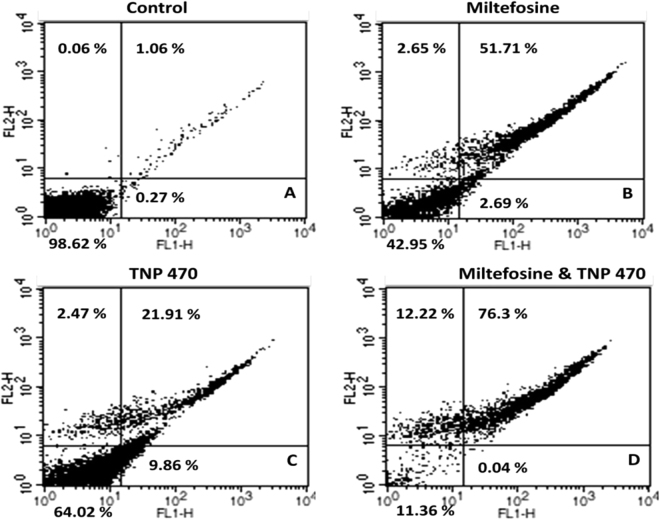
Externalization of phosphatidyl serine on plasma membrane, analysed by annexin V-FITC and PI staining. (**A**) Control promastigotes treated with 0.2% DMSO. (**B**) Promastigotes induced with 25 µM of miltefosine. (**C**) Cells treated with 20 µM of TNP-470. (**D**) Miltefosine induced promastigotes treated with TNP-470.

In higher eukaryotes, cells undergoing apoptosis are characterized by translocation of phosphatidyl serine from the inner side to outer layer of plasma membrane, which can be analyzed by flow cytometry using annexin V-FITC and PI^34^. Miltefosine is well known antileishmanial compound which causes translocation of phosphatidyl serine on outer plasma membrane in case of *Leishmania* promastigotes^31, 32^. A combined use of annexin V-FITC and PI was done to distinguish the apoptotic and necrotic cell death. Annexin V-FITC binds to exposed phosphatidyl serine with high affinity whereas PI selectively enters inside the necrotic cells and binds to DNA which allows the detection of both apoptotic and necrotic cells. Promastigotes treated with 25 µM of miltefosine for 18 h resulted in 51.71% of Annexin-V positive (Fig. 4B), whereas cells treated with 20 µM TNP-470 for 18 h resulted in 21.9% of Annexin-V positive (Fig. 4C). Only 1.06% annexin-V positive cells were present in control promastigotes treated with 0.2% DMSO for 18 h. When the cells were treated with both miltefosine (25 µM) and TNP-470 (20 µM), the Annexin-V positive cells were 76.3% (Fig. 4). Thus, the MAP2 inhibitor along with miltefosine shows cumulative effect for the externalization of phosphatidyl serine on plasma membrane in *L. donovani* promastigotes.

### TNP-470 prevents alteration of transmembrane potential of mitochondria (Δ*Ψm*) caused by miltefosine

**Figure 5:**
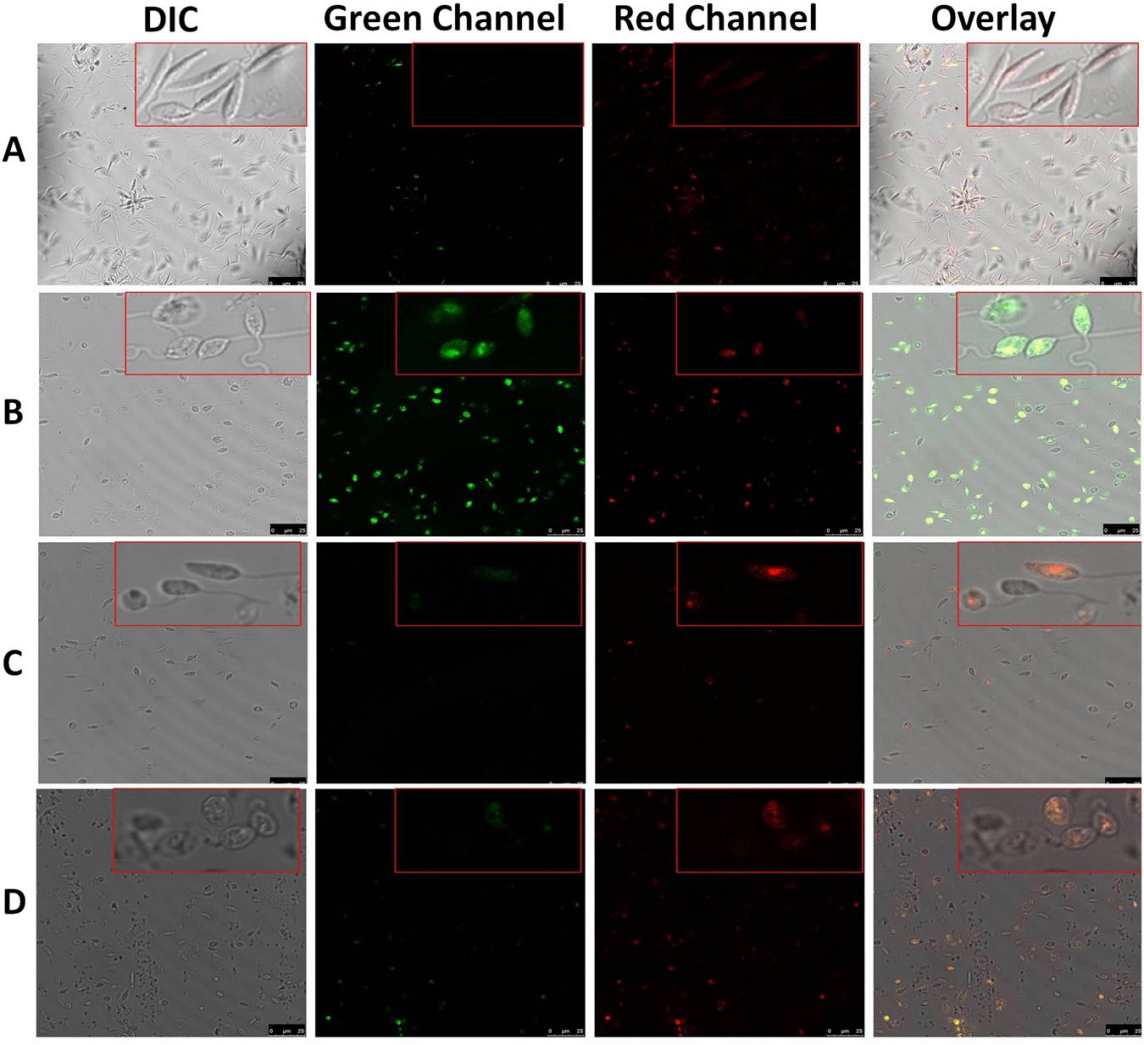
Laser Scanning Confocal Microscopy images of *L. donovani* promastigotes showing the aggregation and monomer forms of Mitocapture^TM^ dye. *L. donovani* promastigotes were treated for 6 hr, stained with Mitocapture^TM^ dye and image was captured by Confocal Microscopy (63x magnification). Increase in red fluorescence indicated the normal mitochondrial membrane whereas increase in green fluorescence showed the alteration in transmembrane potential of mitochondria. (**A**) Control promastigotes treated with 0.2% DMSO. (**B**) Promastigotes induced with 25 µM of miltefosine. (**C**) Cells treated with 20 µM of TNP-470 and (**D**) Miltefosine induced promastigotes treated by TNP-470 for 6 hr. (DIC represents differential interference contrast microscopy).

The depolarization of Δ*Ψm* is the characteristic feature of apoptosis, observed in eukaryotic cells^33^. Change in mitochondrial membrane potential was detected by MitoCapture^TM^ apoptosis detection kit, which is a cationic dye that aggregates in healthy mitochondria giving bright red fluorescence, but at lower membrane potential the dye remains in cytoplasm as monomers that fluoresce green. The mitochondrial membrane potential of promastigotes was examined by confocal microscopy stained by MitoCapture^TM^ dye. Cells were treated with miltefosine and TNP-470, alone or both for 6 hr, mounted on glass-slides and images were captured. As shown in Fig. 5, miltefosine treated cells showed an increase in green fluorescence (Fig. 5B) whereas the TNP-470 treated cells showed an increase in red fluorescence (Fig. 5C). The promastigote cells treated with both miltefosine and TNP-470, showed a significant increase in red fluorescence indicating the role of TNP-470 in the prevention of mitochondrial membrane damage in apoptotic like conditions. Leishmanial cells treated with both miltefosine (25 µM) and TNP-470 (20 µM) for 18 hr incubation at 25 °C, showed maximum debris on glass slides and were not suitable for imaging. Moreover, 6 h time point for mitochondrial membrane potential assay was chosen after optimization and based on earlier report^35^.

### TNP-470 prevents increase in cytosolic calcium caused by miltefosine

**Figure 6:**
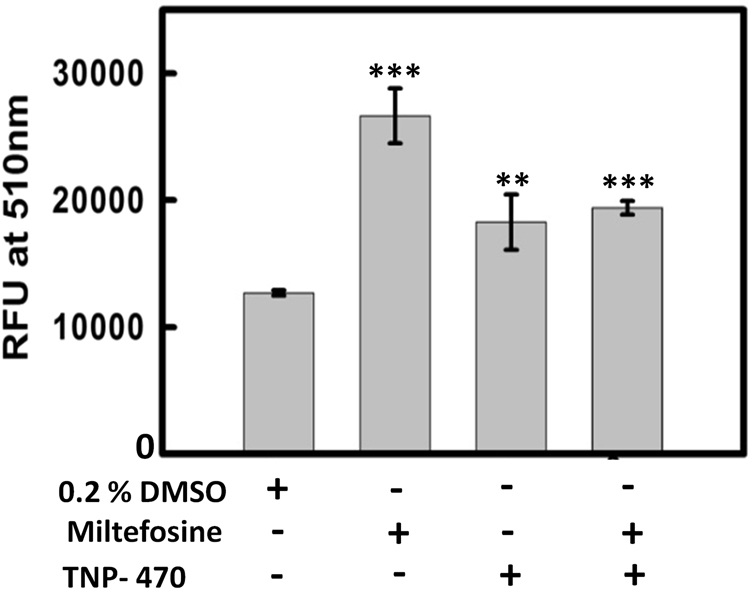
Measurement of Cytosolic concentration of Calcium using FURA2-AM dye, control cells (0.2% DMSO), miltefosine (25 µM) treated promastigotes, TNP-470 (20 µM) treated cells, and promastigote cells treated with both miltefosine and TNP-470 and stained with FURA2-AM dye. Statistical analysis was done using Student’s unpaired t-test in SigmaPlot software (*denotes p value ≤ 0.05, **denotes p value < 0.01 and *** denotes p value < 0.001).

Change in Δ*Ψm* in apoptotic like cell death of parasite results in release of cytochrome C from the mitochondria which in turn enhances the release of calcium from the endoplasmic reticulum, and causes a global increase in cytoplasmic calcium concentration inside the parasite. The increase in cytosolic calcium concentration in miltefosine treated cells was reported by several groups^36, 37^. In our studies, the measured fluorescence intensity at 510 nm was found to be maximum in promastigote cells treated with 25 µM of miltefosine for 18 h (Fig. 6). When cells were treated with both miltefosine (25 µM) and TNP-470 (20 µM) for 18 h, the total cytoplasmic calcium concentration was decreased compared to miltefosine treated cells. The diminished calcium concentration pool after treatment of TNP-470 signifies the retention of Δ*Ψm* as mentioned in above section.

## Discussion

Miltefosine treated *L. donovani* promastigotes shows the characteristic feature of metazoan apoptosis as reported earlier^31, 32^. Several other molecules *i.e* H_2_O_2_^1^, heat shock^38^, staurosporine^39^ etc. are also known to induce PCD in *Leishmania* promastigotes^40^. However, the biochemical pathways responsible for apoptosis like mode of cell death in *Leishmania* are still not fully deciphered*.* In mammalian cells, the proteolytic activation of caspases is essential for the induction of apoptosis^6, 7, 41^. The activation of caspases is also required for the execution of apoptosis in the invertebrates such as *D. melanogaster* ^42^ and *C. elegans* ^43^ *.* Even though the PCD was initially considered in multicellular organisms only, several reports suggested the similar biochemical features of PCD in unicellular eukaryotes such as *T. thermophylia* ^44^, *D. discoideum* ^45^, *T. brucei* ^46^, *T. cruzi* ^47^, *L. major* ^40^, *L. amazonensis* ^38^ and *L. donovani* ^4, 31^.

It is reported that broad caspase inhibitors (BAF) or calpain/cysteine protease inhibitors (E64) prevent the staurosporine induced PCD of *Leishmania* parasite but does not prevent the cell death^40^. Recently, it was reported that the cathepsin B like proteases are involved in PCD of parasite, and are capable to cleave caspase specific substrates^48^. Moreover, calpain inhibitor I was reported to prevent DNA fragmentation more efficiently which can inhibit calpain, cathepsins, cysteine proteases and proteasomes^49^. In contrast, a recent study suggested that the broad spectrum calpain and cysteine protease inhibitor E64 are not able to prevent the miltefosine induced DNA fragmentation. Whereas broad spectrum caspase inhibitor Z-VAD-FMK and Boc-D-FMK prevent the miltefosine induced DNA fragmentation, but have no preventive effect on externalization of phosphatidyl serine^31^. Although the involvements of other cysteine proteases and caspase like activities are reported in PCD of *Leishmania* ^40^, caspase independent cell death pathways are also recognized^50^.

We observed higher level of gene expression of *MAP2* under apoptotic condition, suggesting the importance of *MAP2* protease in PCD of parasite^20^. In the present study, we examined the biochemical effect of TNP-470, a well known MAP2 inhibitor, on the miltefosine induced PCD of parasite. While control *Leishmania* promastigote cells treated with miltefosine showed all characteristics features of apoptosis whereas the *Leishmania* cells treated with both miltefosine and TNP-470 did not show the increased caspase3/7 protease like activity and several other characteristics features of apoptosis. Furthermore, the cell lysates of miltefosine induced promastigotes after pre-incubation with TNP-470, showed the decrease in caspase3/7 protease like activity. It is evident from confocal microscopy data that TNP-470 protects the leishmanial cells from miltefosine induced membrane depolarization as well as increase in the global pool of cytosolic calcium. Further, TNP-470 inhibits the oligonucleosomal DNA fragmentation in miltefosine induced cells. These data together suggests the involvement of MAP2 in PCD of leishmanial cells.

However, TNP-470 treatment of *L. donovani* promastigotes does not inhibit the phosphatidyl serine externalization induced by miltefosine and prevent the cell death. Although, MAP2 inhibition can inhibit induction of apoptosis of parasite under standard apoptotic condition (miltefosine treatment), the MAP2 inhibition is also not favorable for parasite survival as it is also involved in several other key functions. It is worth mentioning that in eukaryotes, MAP2 also plays a significant role in carrying out the regulation of protein synthesis and post translational modifications by removing the N-terminal methionine residue from the nascent peptide and by protection of eIF2α phosphorylation activity. Further, inhibition of phosphatidyl serine externalization is not reported in some other conditions where miltefosine induced apoptosis was inhibited by protease inhibitors^31^. Moreover, one study on *L. donovani* (MHOM/ET/67/HU3) suggests that the Annexin-V binding cannot be a marker of apoptosis in case of *Leishmania* ^51^.

In conclusion, our findings provided the first evidence, to our knowledge that the treatment of *L. donovani* with TNP-470, a well-known MAP2 inhibitor prevented the induction of apoptosis in miltefosine induced promastigotes, but was not able to prevent cell death of the parasites. Based on these observations, our studies suggest the role of MAP2 in *L. donovani*, as an effector molecule in higher eukaryotes which is important for the induction of apoptosis but unable to prevent the cell death. The role of MAP2 in *Leishmania* parasite deserves more attention to understand its role in apoptosis like cell death. Furthermore, studies on mechanism of action of TNP-470 and proteomic analysis may provide the detailed answer.

## Methods

### Parasites, cell lines and chemicals

The *Leishmania donovani* (MHOM/IN/2010/BHU1081) strain was generously donated by Prof. Shyam Sundar, Banaras Hindu University. TNP-470 [5-methoxy-4-(2-methyl-3-(3-methyl-2-butenyl)oxiranyl)-1-oxaspiro(2,5)oct-6-yl (chloroacetyl) carbamate] was obtained from Sigma. Caspase 3/7 Assay kit was procured from Promega. Annexin V-FITC Apoptosis detection kit and MitoCapture^TM^ Apoptosis detection kit was purchased from Calbiochem. All the chemicals used in the experiments were of the highest grade procured from Sigma-Aldrich or Merck.

### Parasite culture and Genomic DNA isolation

*Leishmania donovani* promastigotes were grown at 25 °C in M199 media supplemented with 15% heat-inactivated fetal bovine serum (FBS) and Penicillin streptomycin (1%), and genomic DNA was isolated as reported earlier^4, 52^.

### Cloning, Expression and Purification of *Ld*MAP2

A putative sequence was identified for *Ld*MAP2 from the GeneDB database with accession number LdBPK_210960.1. The coding region of full length *Ld*MAP2 was amplified from *L. donovani* genomic DNA by PCR using the forward primer (*Ld*MAP2 F: AA*GAATTC*ATGCCACCAAAGATGTCTGC), containing an *Eco*RI restriction site and start codon, and the reverse primer (*Ld*MAP2 R: AA*CTCGAG*CTAGTAGTCGCTTCCCTTG), containing *Xho*I restriction site and stop codon. The PCR fragment and pET-28a(+) were digested with *Eco*RI and *Xho*I, then ligated to generate pET-28a-*Ld*MAP2 plasmid. The recombinant construct was transformed into BL21 (DE3) *E. coli* cells for expression of protein. The expressed protein was purified through Ni-NTA affinity chromatography from the pellet containing inclusion bodies and size of the recombinant protein was analyzed on 12% SDS-PAGE.

### Refolding and western blot analysis of *Ld*MAP2

The dialyzed recombinant protein was subjected to refolding. Refolding of Ni-NTA purified *Ld*MAP2 was optimized by testing various buffer conditions with some modifications^53^. The protein was diluted ten times (50 μg protein/ml refolding buffer) followed by drop wise addition of protein in refolding buffer (20 mM Tris-HCl, 300 mM arginine, 20 mM cysteine, 150 mM NaCl, 5% (v/v) glycerol, pH 7.5) at 4 °C. Western blot analysis of purified *Ld*MAP2 was done with antibodies against mouse anti-His (1:1000), and further treated with anti-mouse horse radish peroxidase conjugated secondary antibodies (1:1000). The membrane was further subjected to 3,3′-Diaminobenzidine (DAB) and 1% H_2_O_2_ for immunodetection^54^.

### Determination of *Ld*MAP2 enzyme activity

The fluorogenic substrate L-Methionine 7-amido-4-methylcoumarin (Met-AMC) was used for enzymatic assay. The *Ld*MAP2 enzymatic activity was determined by measuring the release of 7-AMC by fluorescence (λ_Ex_ = 360 nm and λ_Em_ = 440 nm)^55, 56^. *Ld*MAP2 enzymatic assay was carried out at 37 °C with 1 ml reaction volume containing 50 mM Tris-HCl pH 7.5, 5 mM NiSO_4_, 10 µM–150 µM Met-AMC and 15 µg recombinant *Ld*MAP2. The release of 7-AMC was measured after 2 h incubation at 37 °C. The *Km* (Michaelis constant) was determined by an end point assay.

For optimum pH analysis, the buffer systems used were 50 mM Sodium acetate for pH 4.0 to 5.5, 50 mM Sodium phosphate buffer for pH 6.0–6.5 and 50 mM Tris-HCl buffer for pH 7.0–11.0. 5 mM NiSO_4_ was also added in each buffer system. For optimum temperature studies different sets of temperature (20 °C–60 °C) were used. Since *L. donovani* MAP2 is a metalloprotease and its crystal structure complexed with metal ion has not been reported, we have evaluated the activity of *L. donovani* MAP2 with various divalent metal ions. To measure the effect of divalent metal ions on *Ld*MAP2 activity; NiSO_4_, NiCl_2_, CuCl_2_, CaCl_2_, MgCl_2_, ZnCl_2_, MnCl_2_ and EDTA were added at a final concentration of 5 mM in assay mixture.

### *Ld*MAP2 inhibition studies

The known inhibitor of MAP2, TNP-470 was assessed against the recombinant *Ld*MAP2. Single point inhibition assay was carried out in 1 ml reaction volume containing 100 µM inhibitor TNP-470, pre-incubated in 50 mM Tris-HCl buffer pH 7.5, 5 mM NiSO_4_ for 30 min. Varying concentration of substrate Met-AMC and 15 µg recombinant *Ld*MAP2 was added in pre-incubated reaction mixture and further incubated at 37 °C for 2 h. The data were plotted in double-reciprocal plot to examine the mode of inhibition and to calculate the inhibitory constant (*K*_*i*_).

### *In vitro* Cell cytotoxicity assay

MTT [3-(4,5-dimethylthiazol-2-yl)-2,5-diphenyltetrazolium bromide] cell proliferation assay was performed as reported earlier in our publication^57, 58^. The absorbance was measured with microplate reader (BIOTEK Synergy HT) at 570 nm. The color formed is directly proportional to viable cells. Promastigote cells treated with 0.2% DMSO were used as a negative control, whereas IC_50_ value of miltefosine (25 µM) served as positive control. The IC_50_ value of TNP-470 was calculated by plotting percent cell viability vs. concentration.

### Determination of caspase-3/7 protease like activity

Intracellular caspase-3/7 protease like activity in *L. donovani* promastigote cells was measured fluorometrically using Apo-1 homogenous caspase 3/7 activity assay kit (Promega). The assays were performed according to manufacturer’s instructions and as reported earlier^4^. Briefly, 1 × 10^6^ promastigote cells were treated with 20 µM of TNP-470 in presence and absence of miltefosine (25 µM) and incubated for 18 h at 25 °C. 0.2% DMSO treated promastigotes were taken as negative control, whereas 25 µM of miltefosine treated cells were used as a positive control. The release of R110 was measured by fluorescence at an excitation and emission wavelengths of 485 nm and 530 nm, respectively. In parallel set of reactions, caspase3/7 inhibitor was added in reaction mixture prior to the addition of treated cells.

### DNA fragmentation Assay by agarose gel electrophoresis

DNA fragmentation was analyzed by agarose gel electrophoresis of total genomic DNA, which was isolated as reported earlier with minor modifications^4, 52^. Briefly, cell pellets of 1 × 10^8^ promastigotes were lysed in 500 µl of lysis buffer (50 mM Tris-HCl, 10 mM EDTA, 0.5% SDS; pH 7.5) containing proteinase K (100 µg/ml), vortexed and kept overnight to digest at 50 °C. RNase A (0.3 mg/ml) was added and then incubated at 37 °C for 1 h. The lysates were extracted using phenol-chloroform-isoamyalcohol (25:24:1) and centrifuged at 15000 × g for 10 min. The upper aqueous phase was collected, treated with 1/10^th^ volume of 3 M sodium acetate and 2 volume of 100% ethanol overnight at −20 °C. The sample was centrifuged at 15000 × g for 15 min and washed with 500 µl of 70% ethanol. The DNA pellet was dissolved in TE buffer (10 mM Tris-HCl, 1 mM EDTA; pH 8.0) and quantified spectrophotometrically at 260/280 nm. A total of 10 µg of genomic DNA was run on 1.5% agarose gel containing ethidium bromide for 2 h at 50 V and visualized under UV illuminator (Bio-Rad).

### Flow cytometric analysis of DNA content

Flow cytometric analysis of DNA content was done by the methods described in our earlier publications with minor modifications^4^. In brief, 2 × 10^7^ *L. donovani* promastigote cells were treated with 20 µM of TNP-470 in presence or absence of miltefosine (25 µM) for 12 hrs at 25 °C. Cells were then centrifuged at 1000 × g for 5 min and washed twice with cold PBS to remove the traces of media, fixed in pre chilled 70% methanol (added drop wise) and incubated overnight at −20 °C. The fixed cells were centrifuged at 3000 × g, washed twice with cold PBS and then treated with RNase A (200 µg/ml) and incubated at 37 °C for 30 min. Cells were then treated with 20 µg/ml of PI (propidium iodide), incubated at room temperature for 30 min in the dark and acquired by CytoFLEX Flow Cytometer-Beckman Coulter, Inc. The percentage of hypodiploidy was calculated by CytExpert software. The value of Gain for FSC-A was 120 and for SSC-A was 100; Threshold was set to default (automatic); Channel ECD was used for width. ECD-H vs. ECD –A was plotted using SSC vs. FSC data and gate was fixed for all the experiments. ECD-w vs. ECD-A was plotted for gated cells and total number of cells (count) vs. ECD-A was plotted for analysis.

### Laser Scanning Confocal Microscopy analysis to check the transmembrane potential of mitochondria (Δ*Ψm*)

Δ*Ψm* was estimated by using the MitoCapture™ apoptosis detection kit (Calbiochem) according to the earlier reports^35^. Briefly, 1 × 10^7^ promastigote cells after different treatments were centrifuged at 1000 × g for 5 min, washed twice by PBS and resuspended in 1 ml incubation buffer containing MitoCapture™ dye. The cells were incubated for 30 min at 37 °C, washed twice and resuspended into 500 µl of incubation buffer. Stained promastigotes were mounted on a glass slide and were observed by Leica DMi8 Confocal Microscopy under magnification of 63x (1.4 NA). The fluorescence signal was observed sequentially, exciting first at 488 nm (Ar-Kr laser beam) and then at 543 nm (He-Ne laser beam). The green fluorescence was measured at 488 nm excitation and red fluorescence was monitored at 543 nm.

### Detection of phosphatidyl serine exposure on plasmamembrane

Analysis of phosphatidyl serine exposure on plasma membrane of promastigote cells was done by using Annexin-V-FITC apoptosis detection kit (Calbiochem) according to our earlier publications^35, 59^. Briefly, 1 × 10^6^ untreated or treated cells were centrifuged at 1000 x g for 5 min, washed twice with cold PBS and stained with Annexin-V-FITC and PI as per manufacturer’s instructions with minor modifications. The fluorescence intensity was detected by FACSCalibur flow cytometer (Becton Dickinson) and analyzed by CellQuest software.

### Measurement of intracellular Ca^2+^ concentrations

Changes in intracellular Ca^2+^ concentrations were estimated by fluorescent probe, FURA 2AM as described earlier^35^. Cells were treated with TNP-470 and miltefosine as mentioned in above section. Fluorescence intensity was measured at an excitation and emission wavelength of 340 nm and 510 nm, respectively.

## Electronic supplementary material

Fig. S1 and Fig. S2
