# Supplementary material for: Methionine aminopeptidase 2 is a key regulator of apoptotic like cell death in Leishmania donovani
Source: Sci Rep. 2017 Mar 7;7:95. doi: 10.1038/s41598-017-00186-9 (PMC5427942; doi:10.1038/s41598-017-00186-9)
Supplement: Supplementary file 1 — Fig. S1 and Fig. S2 [file 41598_2017_186_MOESM1_ESM.pdf]

Methionine aminopeptidase 2 is a key regulator of apoptotic like cell death in *Leishmaniadonovani*

Ritesh Kumar, Kartikeya Tiwari and Vikash Kumar Dubey*

Department of Biosciences and Bioengineering, Indian Institute of Technology Guwahati, Assam, India- 781039, e-mail: vdubey@iitg.ernet.in

Address for correspondence:

Prof. V.K. Dubey, Department of Biosciences and Bioengineering,
Indian Institute of Technology Guwahati, Assam, India- 781039,
e-mail: vdubey@iitg.ernet.in
Phone No: +91 361 2582203

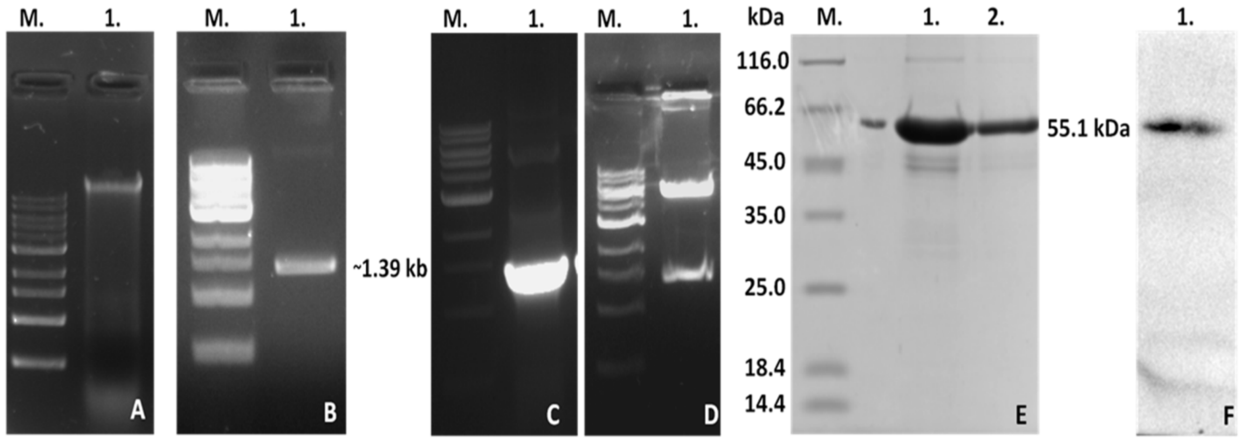

Fig. S1. Sub cloning of *Ld*MAP2 in expression vector pET-28a(+) and expression in BL21 (DE3) *E. coli*. (**A**) Genomic DNA was isolated from *Leishmania donovani*. (**B**) Lane M. 1 kb DNA ladder, Lane 2. PCR amplification of *Ld*MAP2 from genomic DNA of *L. donovani.* **(C)** Clone confirmation of *Ld*MAP2 in pET-28a(+) by PCR. Lane M: 1 kb DNA ladder, lane 1: positive clones. **(D)** Lane M. 1 kb DNA ladder, lane 1. pET-28a-*Ld*MAP2 construct digested with *EcoRI* and *XhoI*. **(E)** SDS-PAGE analysis of purified His-tagged r*Ld*MAP2, Lane M. medium range protein marker, lane 1 and 2. *Ld*MAP2 after Ni-NTA affinity purification. **(F)** Western blot image of Purified His-tagged *Ld*MAP2, mouse anti-His antibodies were used for immunodetection.

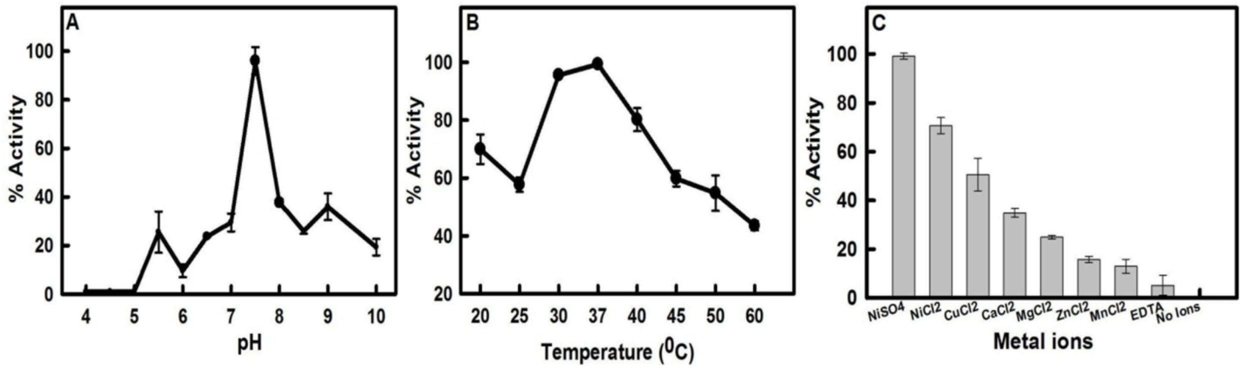

Fig. S2. pH optima, temperature optima and effect of metal ions on enzyme activity of *Ld*MAP2 **(A)** pH- profile. The pH optima studies giving a pH optimum of 7.5. **(B)** Optimum temperature studies. Assays were carried out in different temperature conditions ranging from 20- 60˚C. *Ld*MAP2 has an optimum activity at 37˚C. **(C)** Effect of metal ions on enzyme activity; maximum activity was found in presence of Ni(II) and very less activity was found in presence of EDTA. Data represents the mean ± SD of three independent experiments.
